# Rare paraneoplastic syndromes associated with thymoma: case reports and literature review

**DOI:** 10.3389/fimmu.2026.1948683

**Published:** 2026-10-26

**Authors:** Wenji Jia, Lei Jin, Xicheng Li, Dingxian He, Jie Song, Yiqin Xiao, Yu Xue, Yiqi Wang, Xianglin Chu, Hui Wu, Dongyue Yue, Wenhua Zhu, Jianying Xi, Jing Xiong, Chongbo Zhao, Sushan Luo

**Affiliations:** 1 Department of Neurology, No. 2 Affiliated Hospital, Kunming Medical University, Kunming, China; 2 Huashan Rare Disease Center and Department of Neurology, Huashan Hospital, Shanghai Medical College, National Center for Neurological Disorders, Fudan University, Shanghai, China; 3 Department of Ophthalmology, Huashan Hospital, Fudan University, Shanghai, China; 4 Department of Rheumatology, Huashan Hospital, Fudan University, Shanghai, China; 5 Department of Neurology, Zhejiang Provincial People’s Hospital, Hangzhou Medical College, Hangzhou, Zhejiang, China; 6 Department of Cardiothoracic Surgery, Huashan Hospital, Fudan University, Shanghai, China; 7 Department of Neurology, Jing’an District Centre Hospital of Shanghai, Shanghai, China

**Keywords:** autoimmunity, paraneoplastic pemphigus, paraneoplastic syndromes, rare, thymoma

## Abstract

Thymoma is the most common anterior mediastinal tumor, which is linked to the loss of self-tolerance and the presence of autoimmunity. Various interactions between thymoma and autoimmune diseases have been identified, including myasthenia gravis, systemic lupus erythematosus, pure red cell aplasia, and pemphigus. In this retrospective case series, we reported rare autoimmune disorders with different organ involvement associated with thymoma. These included ocular paraneoplastic pemphigus, neuromyelitis optica spectrum disorder, diffuse panbronchiolitis and myositis. Our series broadens the spectrum of autoimmune disorders with shared T cell-mediated and B cell-dependent pathogenesis, underscoring the recurrence or relapse of thymoma-associated autoimmunity. The recognition of rare autoimmune presentations charts future treatment decisions and multidisciplinary care.

## 1 Introduction

Thymoma is the most prevalent epithelial tumor of the anterior mediastinum. Although approximately 50% of patients remain asymptomatic at the time of diagnosis, thymomas are frequently associated with a broad spectrum of paraneoplastic syndromes (PNS). Myasthenia gravis (MG) constitutes the most common PNS, affecting 25-40% of patients. Other PNS reported in association with thymoma include pure red cell aplasia, lichen planus, or limbic encephalitis (1, 2). In a multicenter review of 85 thymoma patients treated between 2005 and 2011, 55% of patients presented with one or more immunological disorders (3).

The pathogenesis of PNS encompasses both antibody- and non-antibody-mediated mechanisms, with approximately one-third of affected patients exhibiting multisystem involvement (4). PNS may develop either before or after thymectomy, although the occurrence of two or more PNS in the same individual-whether concurrent or sequential-is relatively uncommon. In our previous cohort study, the presence of infectious complications and multiple PNS was significantly associated with poor overall survival, highlighting the clinical importance of their early recognition (5). Therefore, prompt identification of rare PNS is essential for predicting prognosis and refining risk stratification in patients with thymoma.

In this retrospective case series, we report rare presentations of multiple PNS in patients with a history of thymoma, including paraneoplastic pemphigus (PNP), neuromyelitis optica spectrum disorder (NMOSD), inflammatory myopathy, interstitial lung disease, exfoliative dermatitis, and diffuse panbronchiolitis (DPB). By detailing their clinical evolution, immunological profiles, and therapeutic responses, we aim to enhance the recognition of the clinical manifestations of thymoma-associated autoimmunity (**Table 1**).

**Table 1:** A summary of cases with thymoma presenting multiple autoantibodies and rare paraneoplastic syndromes. .

No.	Thymoma pathology	Recurrence	Autoimmune manifestations	Antibody status	Treatments	Outcomes
1	Masaoka I, T2N0M0	No	Ocular PNP (5 years after thymectomy); MG	Anti-DSG1 antibody (+), anti-AChR antibody (+)	IVIG/high-dose steroids	Improved
2	Masaoka II, T2N0M0	Yes	NMOSD; Exfoliative dermatitis; DPB (12 years after thymectomy); MG	Anti-AQP4 antibody (+),anti-AChR antibody (+)	IVIG/anti-microbial treatments/C5 inhibitors (eculizumab)	Dermatological symptoms and NMOSD improved; Died of recurrent pneumonia and respiratory/circulatory failure
3	Masaoka II, T1aN0M0	No	ASS; myositis (post-thymectomy)	Anti-Jo-1 antibody (+), anti-Ro-52 antibody (+)	PE/high-dose steroids/IVIG/immunosuppresssants	Improved

PNP, paraneoplastic pemphigus; MG, myasthenia gravis; DSG, desmoglein; AChR, acetylcholine receptor; IVIG, intravenous immunoglobulin; NMOSD, neuromyelitis spectrum disorders; DPB, diffuse panbronchiolitis; AQP4, aquaporin 4; ASS, anti-synthetase syndrome; PE, plasma exchange.

## 2 Case description

### 2.1 Case 1 - ocular paraneoplastic pemphigus and Myasthenia gravis

A 71-year-old male with 7-year ptosis, limb weakness, and skin rash presented dyspnea in 2019. Elevated serum anti-acetylcholine receptor (AChR) (12.60 nmol/L; ref <0.5), anti-Titin (++), and anti-RyR (++) antibodies, plus CT-detected thymoma, led to intravenous immunoglobulin (IVIG)/methylprednisolone and thymectomy (WHO A, Masaoka I, T1aN0M0). Postoperatively, myasthenic crisis (MC) and extensive blisters occurred; skin biopsy confirmed pemphigus-like pathology. After repeated cycles of IVIG/high-dose steroids, ventilator weaning was enabled. In 2024, right eye redness/pain/corneal opacities (misdiagnosed as conjunctivitis) led to amniotic membrane transplantation, later attributed to PNP (**Figure 1**). Ophthalmic examination showed right eye only light perception with subconjunctival hemorrhage, corneal ulceration/staphyloma, anterior chamber loss, corneal and lens opacities (no keratic precipitates); left eye had nebula, epithelial defect, contact lens, and lens opacity. Serum anti-desmoglein 1 (DSG1) antibody level was markedly elevated (171.2 U/ml; ref <14).

**Figure 1:**
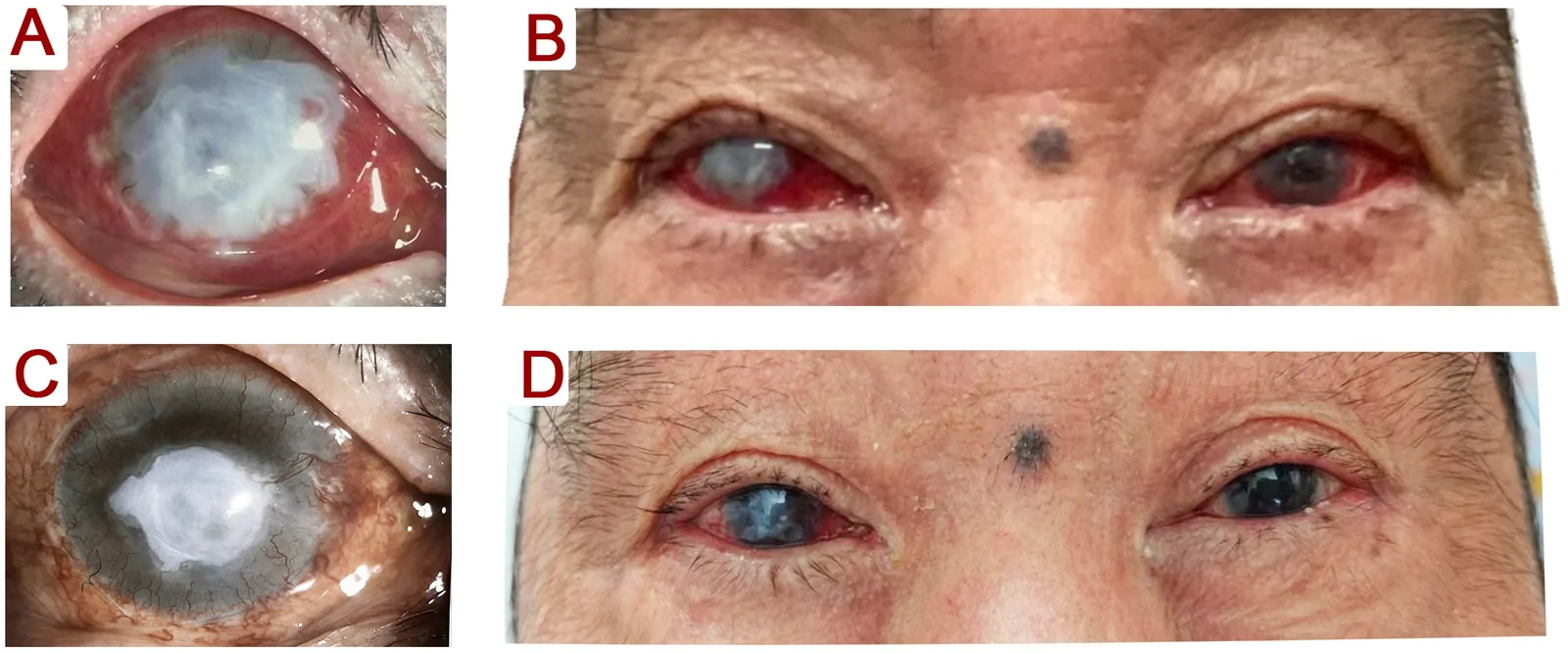
Paraneoplastic pemphigus affecting the conjunctiva and cornea. **(A, B)** Conjunctival hyperemia and corneal ulceration in the right eye developed 5 years after thymoma resection. **(C, D)** After treating with three cycles of intravenous immunoglobulin (0.4g/kg/d for 5 days for each cycle), conjunctival hyperemia was relieved while the corneal ulcer significantly improved.

Diagnosis: 1. Ocular PNP; 2. MG (generalized MG, AChR antibody-positive); 3. Thymoma (WHO A, Masaoka I).

### 2.2 Case 2 - neuromyelitis optica spectrum disorder, exfoliative dermatitis and diffuse pan bronchiolitis

A 41-year-old woman presented with a 14-year history of fluctuating ptosis and diplopia, accompanied by a 1-week history of fever and productive cough. In 2011, she was diagnosed with ocular MG based on fluctuating symptoms, a positive neostigmine test, and detectable anti-AChR antibodies. She underwent thymomectomy, with pathology confirming a type B2 thymoma (WHO B2, Masaoka stage II, T2N0M0). Postoperatively, she developed a MC, which resolved with high-dose intravenous methylprednisolone. Long-term immunosuppression was maintained with prednisone, pyridostigmine, and tacrolimus. In February 2023, she developed a generalized erythematous exfoliative rash (exfoliative dermatitis), and concurrent imaging revealed tumor recurrence, prompting radiotherapy.

In June 2023, she presented with right lower limb weakness and numbness. Cervical MRI showed intramedullary signal heterogeneity with focal high T2 signal at C2–C3 and C5–C6, and thoracic MRI revealed similar lesions at T3 and T7 (**Figures 2C, D**). Immunofluorescence staining of the serum sample revealed a positive result for anti-aquaporin-4 (AQP4) antibody (1:32), which led to the diagnosis of NMOSD. In December 2023 and January 2024, she received additional IVIG and satralizumab (an IL-6 receptor antagonist) for NMOSD relapse. However, she had recurrent bronchiolitis, and CT showed diffuse small airway bronchiectasis of centrilobular distribution, with possible “tree-in-bud” appearance. These radiological findings, together with the history of persistent cough and nasosinusitis, led to the diagnosis of diffuse panbronchiolitis (DPB) (**Figures 2A, B**). Sputum culture grew multidrug-resistant *Pseudomonas aeruginosa*, treated sequentially with piperacillin-tazobactam, meropenem, and ceftazidime-avibactam per susceptibility. She also developed *Clostridioides difficile* colitis, managed with vancomycin. MG subsequently relapsed, and given persistent anti-AChR antibodies, eculizumab was initiated. Ultimately, she succumbed to recurrent pulmonary infections complicated by respiratory and circulatory failure.

**Figure 2:**
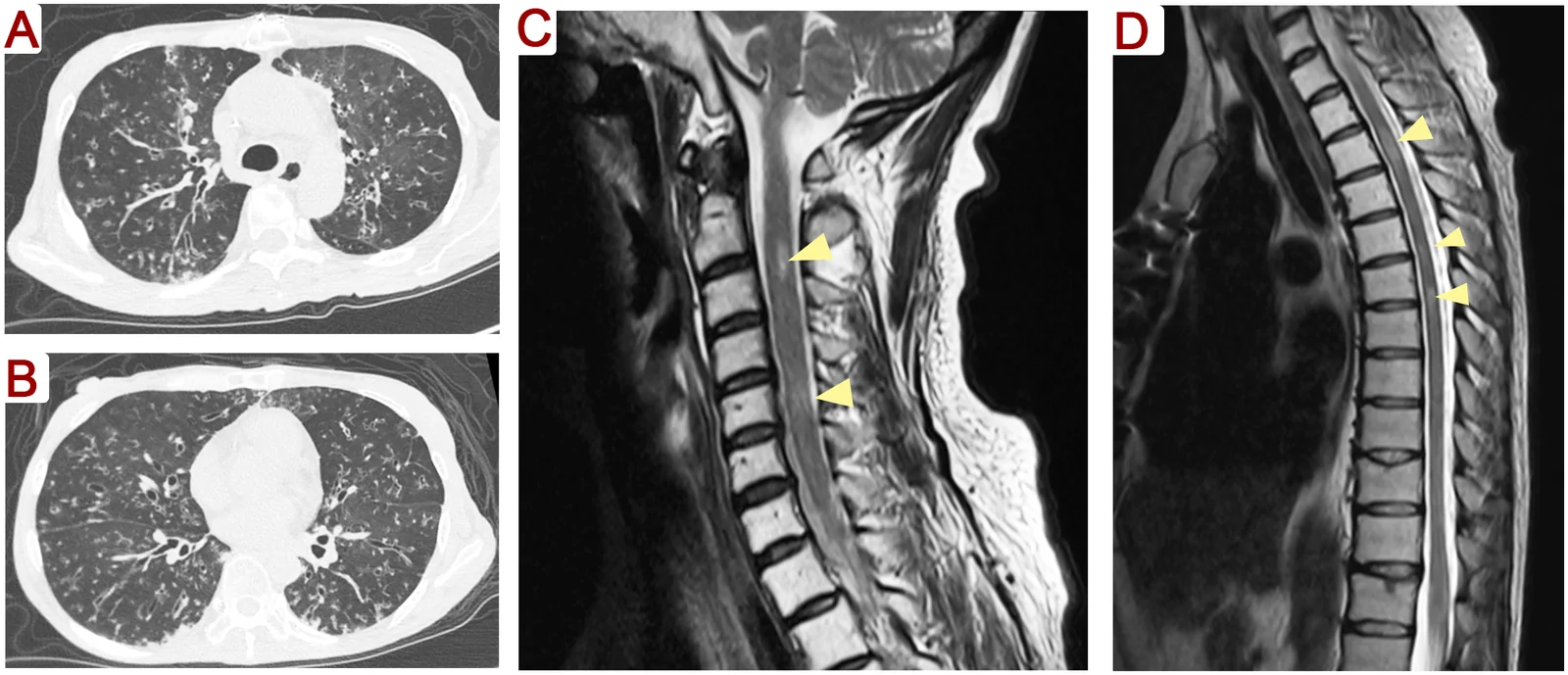
Diffuse panbronchiolitis and neuromyelitis optica spectrum disorder. **(A, B)** Chest CT showing diffuse small airway bronchiectasis of centrilobular distribution, with possible “tree-in-bud” appearance, developed at 12 years after thymectomy. **(C)** Cervical MRI showing small patchy hyperintensities in the spinal cord on T2-weighted imaging at the lower margin of cervical C2-C3 and C5-C6 (yellow arrow). **(D)** Thoracic MRI showing small patchy hyperintensities in the spinal cord on T2-weighted imaging at thoracic T3 and T7 (yellow arrow).

Diagnosis: 1. NMOSD; 2. Exfoliative dermatitis; 3. DPB; 4. MG; 5. Thymoma (WHO B2, Masaoka II).

### 2.3 Case 3 - anti-synthetase syndrome,myositis

A 70-year-old hypertensive male presented with 5-year exertional dyspnea and 13-day bilateral arm pain, with recurrent dyspnea since 2019. In July 2024, he had atrial fibrillation (AF), markedly elevated creatine kinase (CK) (8449 U/L; ref <200), positive anti-Ro-52 and Jo-1 antibodies, and Holter-documented persistent AF (longest R-R interval 2.13s); echocardiography showed normal LV function. Chest CT revealed anterior mediastinal mass, interstitial pneumonia, and effusion. Thigh MRI showed muscle/fascial edema (**Figure 3B**), and muscle biopsy confirmed myositis features: perifascicular necrosis with CD8^+^ T-cells (**Figures 3C, F**).

**Figure 3:**
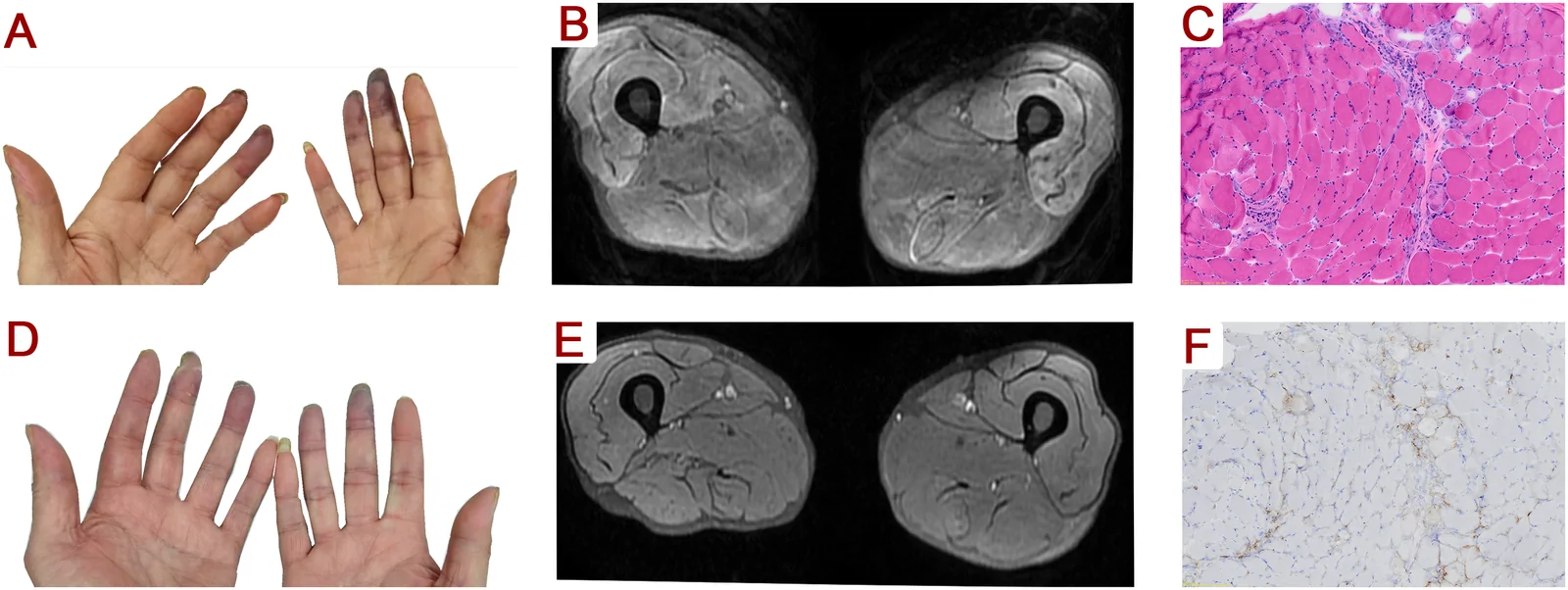
Raynaud’s phenomenon and inflammatory myopathy. **(A)** Images showing purple discoloration and hardening of the skin at the fingertips after thymectomy. **(D)** After treatment with intravenous immunoglobulin and methylprednisolone, skin color normalized and texture softened. **(B)** Thigh MRI showing bilateral muscle and fascial edema in short Tau Inversion Recovery sequence. The edema greatly improved post-treatment **(E)**. **(C)** Muscle pathology findings, showing perifascicular degeneration and necrosis of muscle fibers with phagocytic reactions after HE staining (200×); and **(D)** perivascular CD8-positive cell infiltration in fascial layers (200×).

In August 2024, thoracoscopic thymoma resection was performed, and pathology confirmed WHO B3 (Masaoka II, T1aN0M0) thymoma. Postoperatively, acute respiratory failure and limb weakness required mechanical ventilation; he received plasma exchanges and methylprednisolone, then was weaned and discharged. Three days after stopping oral immunosuppressants in December 2024, he developed digital burning pain, swelling, cyanosis, and numbness (Raynaud’s phenomenon, **Figure 3A**), without motor deficits. IVIG and resumption of immunosuppressants led to marked symptom improvement. **Figures 3D, E** presented the fingers and thigh muscle imaging after immunotherapies.

Diagnosis: 1. Anti-synthetase syndrome (ASS); 2. Thymoma (WHO B3, Masaoka II); 3. Myositis.

## 3 Discussion

Thymoma is a rare epithelial neoplasm with diverse histology and clinical behavior. The thymus sustains T-cell maturation and immune activity into adulthood, predisposing to autoimmune disorders after thymectomy—including neurological, hematological, and dermatological conditions (6). Our series broadens this spectrum with PNP, NMOSD, DPB, and myositis, underscoring that thymoma-associated autoimmunity is a systemic dysregulation necessitating integrated management.

### 3.1 Thymoma and PNP

PNP is a rare autoimmune disorder with a mortality rate approaching 90% (7), characterized by severe mucocutaneous erosions and, in some patients, ocular and pulmonary involvement (8, 9). Although PNP is most linked to lymphoproliferative malignancies, thymoma is a recognized solid tumor association (10). To date, only 10 cases of ocular PNP have been described in detail (8, 11, 12), underscoring the importance of early diagnosis to prevent delayed malignancy detection.

Our case met all diagnostic criteria for PNP and uniquely presented concurrently with myasthenic crisis. This association is mechanistically supported by cross-reactivity between tumor antigens (e.g., thymic epithelial antigens) and skin/mucosal proteins (13). Although B-cell depletion is recommended for hematological malignancy-associated PNP (14), it was contraindicated in our patient due to concurrent infections. Multimodal therapy improved skin, mucosal, and ocular lesions, offering an alternative strategy for thymoma-associated PNP. Early tumor resection remains the backbone of the therapies, though residual symptoms such as oral ulcers may persist beyond two years (8). For ocular involvement, biopsy of accessible sites (e.g., oral mucosa) rather than ocular tissues is recommended (9), along with mandatory malignancy screening. Despite aggressive management, prognosis remains poor, largely due to obliterative bronchiolitis or sepsis (7). In summary, PNP is a rare, life-threatening disease that should be considered in patients with refractory conjunctivitis or eyelid ulceration.

### 3.2 Thymoma and NMOSD

NMOSD following thymoma arises from thymus-mediated cross-reactive immune responses and postoperative immune imbalance. Multidisciplinary collaboration of neuroimmunology and thoracic surgery is essential for early identification and individualized immunomodulatory strategies. Approximately 3–25% of NMOSD cases may be paraneoplastic, with neurological symptoms triggered by an underlying malignancy before or after cancer diagnosis (15). NMOSD is primarily an antibody-mediated astrocytopathy, and AQP4 expression in thymoma tissue provides a plausible biological link to paraneoplastic NMOSD (16, 17). Other solid tumors, including breast and lung cancers, have also been associated, though their incidence requires validation in large cohorts (18).

While thymectomy can alleviate thymoma-associated MG, it may exacerbate immune imbalance. Postoperatively, 43% of patients develop new autoantibodies (e.g., ANA) and 12.5% develop new autoimmune diseases. Delayed-onset NMO, with latency extending to several years, may result from residual AQP4-reactive T cells migrating to peripheral lymphoid tissues and becoming activated by skeletal muscle injury or other triggers (19). In an analysis of 70 cases with coexisting thymoma and NMO, 70% of patients achieved MG remission after thymectomy, but NMO continued to progress; anti-AChR antibody levels declined while anti-AQP4 antibody levels increased, suggesting distinct pathological mechanisms (20). B-cell depletion agents such as rituximab can target both MG and NMO humoral pathways.

Further research is needed to explore the T-/B-cell interaction network within the thymoma microenvironment and the molecular triggers of AQP4-specific T-cell activation, such as the IL-21/IL-10 axis (21). In our case, the patient developed recurrent spinal cord and brainstem symptoms with AQP4 antibody positivity post-thymectomy, accompanied by immune deficiencies suggested by recurrent infections—consistent with this pattern of immune dysregulation. Although some dermatological and neuroimmunological manifestations have been partially reported previously (22), the present study provides clinical associations and hypothesis-generating observations.

Currently, although the PNS-Care criteria classify AQP4-IgG as a low-risk paraneoplastic antibody (tumor association <5%) (23), its paraneoplastic potential remains underestimated. In thymoma or MG patients presenting with myelitis, optic neuritis, area postrema symptoms, or unexplained brainstem manifestations, AQP4-IgG testing and spinal cord MRI should be performed promptly. Conversely, thymoma screening may be warranted in selected NMOSD patients with MG, other autoimmune manifestations, or atypical systemic features. NMOSD following thymoma arises from thymus-mediated cross-reactive immune responses and postoperative immune imbalance. Multidisciplinary collaboration between neuroimmunology and thoracic surgery is essential for early identification and individualized immunomodulatory strategies.

### 3.3 Thymoma and DPB

Pulmonary involvement was another key feature in our series. DPB is rarely associated with thymoma, with only 30 cases reported globally as of June 2025 (24, 25). Among these, 53.33% (16/30) were type B thymomas, and 36.67% (11/30) were complicated by Good’s syndrome and recurrent infections.

DPB pathogenesis involves significant CD8^+^ T-lymphocyte infiltration, with a reduced CD4^+^/CD8^+^ ratio in bronchoalveolar lavage fluid (BALF) serving as a key immunological hallmark (26, 27). This immune profile aligns with thymoma-induced T-cell dysregulation. Although the relative contributions of thymoma, thymectomy, and radiotherapy to cellular immunodeficiency remain debated (28, 29), our case—with DPB coexisting alongside MG, exfoliative dermatitis, and NMOSD—underscores the central role of T-cell depletion. Post-thymectomy reductions in CD4^+^ lymphocytes may exacerbate immune dysfunction, promoting Good’s syndrome and DPB.

In thymoma patients presenting with persistent cough, sputum, dyspnea, or sinusitis, evaluation for small-airway disease and immune deficiency should be considered. Typical DPB imaging findings include diffuse centrilobular nodules and a “tree-in-bud” pattern. Dynamic monitoring of T-cell subsets before and after thymectomy or radiotherapy is crucial for identifying progressive immunodeficiency. Early macrolide therapy, administered in 17 of 18 literature-reported cases, can prevent irreversible bronchiectasis (30), supporting systematic screening of high-risk patients. Comprehensive assessments integrating pulmonary function tests, CD4^+^ monitoring, and timely macrolide use can significantly improve outcomes.

### 3.4 Thymoma and idiopathic inflammatory myopathies

IIM complicates approximately 0.5-9% of thymoma cases (31). Thymoma is present in up to 77% of IIM patients with MG, markedly higher than the 15% prevalence in isolated MG (32). However, concurrent MG and myositis is rare: among 101 MG patients, only 9.9% had elevated CK, and only one was confirmed to have myositis (33). When thymoma-associated MG is complicated by myositis, limb weakness is common, and bulbar or respiratory muscle involvement may precipitate respiratory failure (31).

In our patient, cardiac manifestations, elevated CK, anti-Ro-52 positivity, myogenic electromyography, and muscle biopsy findings supported inflammatory myopathy alongside MG. These observations highlight the need to screen for thymoma and myositis in patients with atypical muscle weakness, particularly when fluctuating weakness is accompanied by CK elevation, myocarditis-like features, or poor response to standard MG therapy.

Several anti-ARS antibodies have been reported, including anti-Jo-1, PL-7, PL-12, EJ, OJ, KS, Zo, and Ha (34). Anti-Jo-1 is the most common, followed by anti-EJ, anti-PL-7, and anti-PL-12. Some studies suggest that dynamic changes in anti-Jo-1 levels correlate with disease activity (35). Skeletal muscle pathology remains a diagnostic cornerstone, with muscle biopsy being particularly valuable in atypical presentations (36).

## 4 Conclusions

Our cases reveal that thymoma-associated PNS extend beyond the spectrum of MG. The rare coexistence of multiple autoimmune diseases in individual patients suggests the presence of a unique immune microenvironment, which cannot be explained solely by demographic or environmental factors. However, the limitation of this case series included the selectively reported sample, retrospective design and the absence of the control group. Further research is urgently needed to explore the genetic susceptibility and molecular mechanisms driving multi-organ autoimmunity during thymoma development.

## Data Availability

The original contributions presented in the study are included in the articlesupplementary material. Further inquiries can be directed to the corresponding authors.
